# Reliability of hemodynamic parameters measured by bioimpedance cardiography at different intensities during incremental exercise testing

**DOI:** 10.3389/fcvm.2025.1531027

**Published:** 2025-04-10

**Authors:** Raphael Schoch, Jonathan Wagner, Max Niemeyer, Fabienne Bruggisser, Denis Infanger, Justin Carrard, Benedikt Gasser, Arno Schmidt-Trucksäss, Raphael Knaier

**Affiliations:** ^1^ Department of Sport, Exercise and Health, Division Sports and Exercise Medicine, University of Basel, Basel, Switzerland; ^2^ Department Medicine, Training and Health, Institute of Sport Science and Motology, Philipps-University Marburg, Marburg, Germany; ^3^ Department of Clinical Research, University of Basel, Basel, Switzerland

**Keywords:** hemodynamic parameters, stroke volume, impedance cardiography, reliability, peak oxygen uptake

## Abstract

**Background:**

Bioimpedance cardiography offers a non-invasive and time-efficient method to measure hemodynamic parameters. Previous studies only investigated its reliability under steady-state conditions and at maximum load but not at ventilatory thresholds (VTs). This is the first study that assesses the reliability of measured hemodynamic parameters at different exercise stages during cardiopulmonary exercise testing (CPET) using prespecified strict criteria to assess reliability.

**Methods:**

Data from 31 healthy, well-trained adults were analyzed. Each participant completed two CPETs, both following the same ramp protocol, with a 7-day interval between them. Hemodynamic parameters were measured with the PhysioFlow® (Manatec Biomedical, Poissy, France) at characteristic phases and thresholds [VT1, VT2, and peak oxygen uptake (V̇O_2peak_)]. To ensure comparability, the wattage (power) corresponding to the thresholds in Test 1 (P_VT1_, P_VT2_, and P_V̇O2peak_) was used for Test 2.

**Results:**

Heart rate, stroke volume, and cardiac output demonstrated good reliability on a group level (mean intraclass correlation >0.75) at both thresholds (0.91, 0.80, and 0.77 at P_VT1_; 0.92, 0.80, and 0.77 at P_VT2_) and at P_V̇O2peak_ (0.93, 0.82, and 0.80). For stroke volume at P_V̇O2peak_, both individual differences (−39.0 to 36.9 mL for the women and −39.9 to 45.2 mL for the men) and mean detectable change (17.5 mL) were larger than the *a priori* defined acceptable ranges of agreement (−3.6 to 3.8 mL for the women and −4.5 to 3.3 mL for the men).

**Conclusion:**

The PhysioFlow® reliably measures heart rate, stroke volume, and cardiac output during CPET on a group level. However, as shown by the Bland–Altman plots, the reliability is too low to be used for individual comparisons.

## Introduction

Cardiac output (CO) is important in clinical settings to understand cardiac function and hemodynamics. In addition to electrocardiogram, ventilation, and gas exchange, CO provides useful diagnostic information on cardiac function during exercise and helps to evaluate factors limiting exercise capacity (1–4). This information enables more precise treatment of patients in clinical settings (5, 6).

The gold standard to assess CO is the highly invasive direct Fick method, which is expensive and requires adherence to stringent conditions for accurate measurements (7). Thus, this method is less suitable for clinical routine (8). In addition to the direct Fick method, several methods are available to measure CO (9, 10). The transthoracic impedance cardiography method is one of them, which uses electrodes on the neck and chest to detect and transmit impedance changes in the thorax to calculate hemodynamic parameters (11). Impedance cardiography offers a non-invasive, economical, and fast method of measuring hemodynamic parameters during exercise (12). The Physioflow® device uses transthoracic impedance analysis to measure CO (13). Compared to the direct Fick method, it demonstrates an acceptable correlation (*r* = 0.85) during steady-state exercise at low workloads (10–50 watts), which were selected based on the participants’ fitness levels to remain below the ventilatory threshold (VT) and ensure steady-state conditions (14), and a high correlation (*r* = 0.94) at high-intensity exercise (15). However, a high correlation does not necessarily indicate good validity, as demonstrated by the mean difference and 95% confidence interval (CI_95%_) in the Bland–Altman comparison between the direct Fick and the transthoracic impedance method, which was 2.89 L/min (−4.04; 2.89) or, as described in a percentage, −11% (−26.94%; 21.38%).

The reliability of the PhysioFlow® impedance cardiograph has been investigated during rest (13, 16) and at low to moderate intensity under steady-state exercise (13, 14), but limited data are available at high-intensity and maximum loads (13, 17, 18). Only one study examined reliability at different stages during an incremental exercise test (15). In clinical settings, VTs are used to assess exercise and functional capacity, especially in submaximal areas (19). Thus, if CO can be reliably measured at VT and peak exercise, it could benefit diagnosis, risk stratification, and treatment strategies, especially for cardiovascular diseases (20). This study aimed to assess the reliability of the PhysioFlow® device at the VTs during cardiopulmonary exercise testing (CPET), which is still unknown.

The second innovative approach of this study is how reliability is defined. In previous studies, there was disagreement about what represents acceptable reliability for this device. While some studies considered a ±30% agreement limit acceptable (21), later studies using Bland–Altman plots considered limits of agreement with a 95% confidence interval (CI_95%_) of less than ±20% acceptable (15). Other studies (22) defined an intraclass correlation (ICC) coefficient of ≥0.7 as reliable. In contrast, this study is the first to set strict data-driven and *a priori*-defined criteria to classify reliability by using the lower limits of the CI_95%_ of the ICC and calculate the effect of stroke volume (SV) on peak oxygen uptake (V̇O_2peak_). Such rigorous reliability assessment is crucial for the routine use of impedance cardiography, thus, this study assesses the reliability of the standard hemodynamic parameters provided by the PhysioFlow® [heart rate (HR), SV, and CO] at different stages (at VT1, VT2, and V̇O_2peak_) during CPET in healthy, well-trained adults. In addition, the reliability of all other parameters provided by the PhysioFlow® was investigated, including the SV index (SVI), cardiac index (CI), ventricular ejection time (VET), contractility index (CTI), left cardiac work index (LCWi), systemic vascular resistance index (SVRi), systemic vascular resistance (SVR), early diastolic filling ratio (EDFR), end-diastolic volume (EDV), and ejection fraction (EF).

## Methods

### Study population

**Figure 1:**
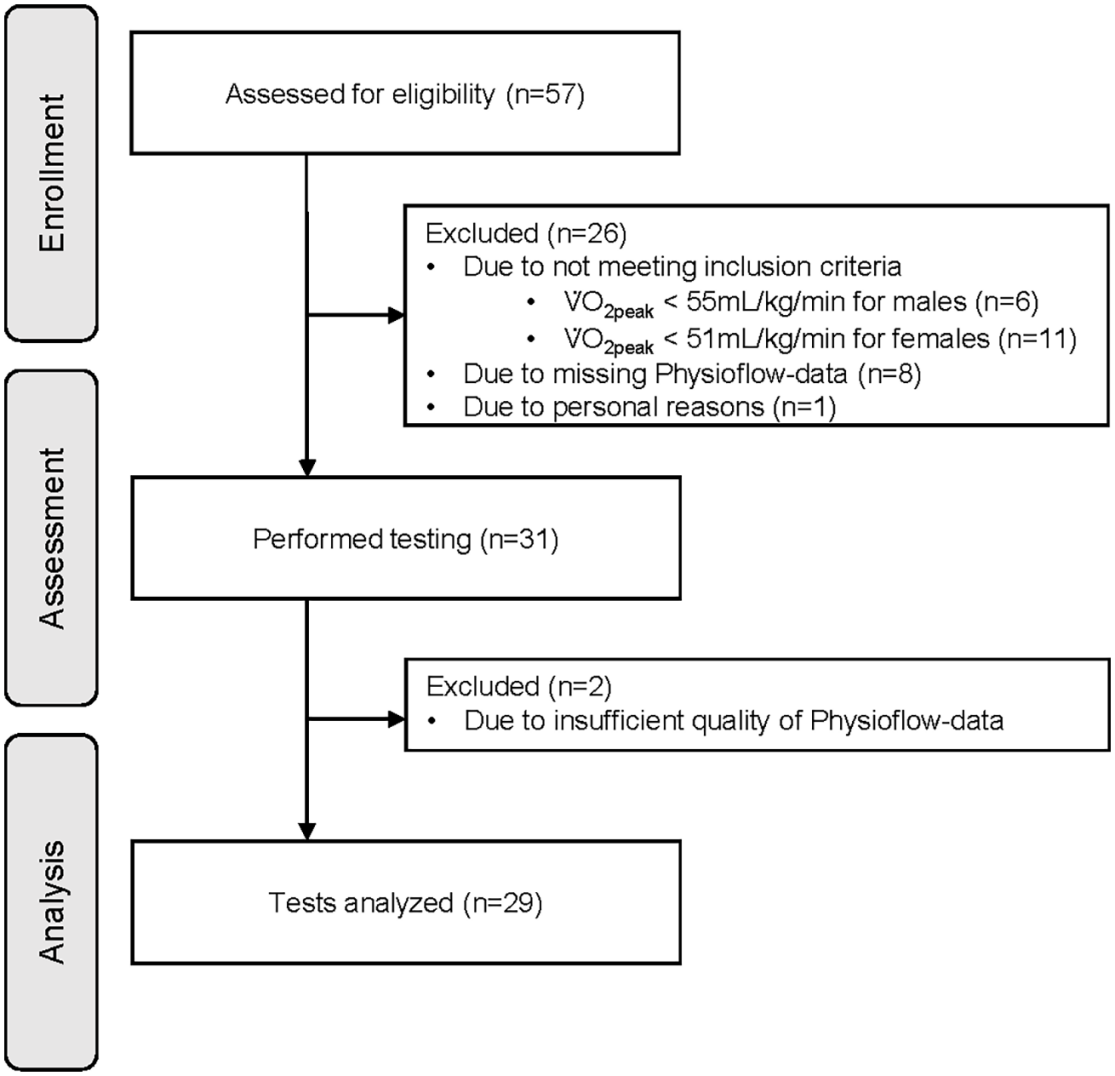
Flow diagram representing enrollment, assessment, and analysis of participants. V̇O_2peak_, peak oxygen consumption.

In total, 57 healthy and well-trained adults participated in this study. The original sample size was determined based on a power analysis for a primary outcome investigated in a separate study. Since this study focused on a secondary outcome, no specific power calculation was performed. Inclusion criteria were age between 18 and 39 years, body mass index ≤ 27 kg/m^2^, and V̇O_2peak_ ≥ 51 mL/kg/min for women and ≥ 55 mL/kg/min for men. The criterion for V̇O_2peak_ was based on the 95th percentile according to the normative data of the American College of Sports Medicine references (23). V̇O_2peak_ was measured during the first CPET and participants who did not reach the required V̇O_2peak_ values were excluded. Exclusion criteria were a history of cardiovascular events, the presence of cardiovascular diseases, type 2 diabetes, pregnancy, or febrile infection within the last 14 days. A total of 26 participants did not meet the inclusion criteria and were excluded, resulting in 31 participants for analysis (Figure 1). The study was approved by the ethics committee “Ethikkommission Nordwest- und Zentralschweiz” (EKNZ: 2019-01697). All participants participated voluntarily and signed written informed consent.

### Cardiopulmonary exercise testing

Two CPETs with an identical ramp protocol were performed by the participants, with a recovery period of 7 days between tests (24). To ensure equal testing conditions, CPETs were performed at the same time of the day (25). Subjects were asked to eat and drink water adequately 72 h before each test and abstain from strenuous activity and alcohol for 24 h, caffeine for 4 h, and food for 2 h before each test (24).

The tests were performed on a cycle ergometer (Sport Excalibur, Lode Medical Technology, Groningen, The Netherlands) using a fixed ramp protocol (3 min rest, 3 min warm-up at 50 Watts, linear increase of 30 W/min until exhaustion, 3 min cool down at 50 Watt) (26). All measurements were performed under standardized conditions (temperature 20°C–22°C, air humidity 40%–55%). Saddle and handlebar positions were individually adjusted on the first day (Test 1) and set the same for the second day (Test 2). Ventilated air volume and expired gas concentrations were measured via breath-by-breath analysis (MetaMax 3B, Cortex Biophysik GmbH, Leipzig, Germany) and calibrated before each test. HR was measured with 12-channel electrocardiography (Custo med GmbH, Ottobrunn, Germany). On the first visit before the CPET, a physician monitored and controlled the electrocardiography, and all participants filled out the physical activity readiness questionnaire for safety reasons (27).

### Determination of VT1, VT2, and V̇O_2peak_

VT1 was evaluated according to the three validated methods described by Binder et al. (28): (1) V̇-slope method [deflection point in the relationship between carbon dioxide production (V̇CO_2_) and V̇O_2_]; (2) ventilatory equivalent method [first increase in ventilatory equivalent for oxygen (V̇_E_/V̇O_2_) with remaining ventilatory equivalent for carbon dioxide (V̇_E_/V̇CO_2_)]; and (3) end-tidal oxygen pressure/workload method (increase in end-tidal oxygen pressure). VT2 was determined with three methods described by Anselmi et al. (29): (1) V̇_E_/V̇CO_2_ plot [disproportional increase of minute ventilation (V̇_E_) vs. V̇CO_2_]; (2) end-tidal CO_2_ pressure/workload (deflection point of end-tidal CO_2_ pressure); and (3) V̇_E_/workload relationship (lowest point of relationship). Two independent investigators determined VT1 and VT2. In cases of disagreements between the two investigators, a third investigator (RS) was involved in the process. V̇O_2peak_ was defined as the highest consecutive 30 s of V̇O_2_ at any point during the test and a respiratory exchange ratio ≥1.13 (30, 31). VT1, VT2, and V̇O_2peak_ were determined in Test 1. To analyze the reliability of the hemodynamic parameters between both tests, the wattage at VT1, VT2, and V̇O_2peak_ in Test 1 was measured and defined as P_VT1_, P_VT2_, and P_V̇O2peak_. For Test 2, the same wattage at each threshold was used. This ensures that the test-retest reliability was examined under the same physiological conditions rather than the reliability of VTs and V̇O_2peak_.

### Hemodynamic monitoring by impedance cardiograph

Hemodynamic parameters were measured with the non-invasive impedance cardiograph PhysioFlow® Endure^TM^ (PhysioFlow®, Manatec Biomedical, Poissy, France). The operating principle has been described in detail by Charloux et al. (14). The PhysioFlow® operates with changes in transthoracic impedance during cardiac ejection to calculate the SVi. Before attaching the electrodes (PhysioFlow HTFS50PF, Manatec Biomedical, Poissy, France), the skin was prepared according to the manufacturer's recommendations (shaved, disinfected, and cleaned with abrasive gel). Two electrodes were used to measure a 1-channel-electrocardiography (position V1/V6). Another four electrodes were placed pairwise on the left side of the neck, between the earlobe and clavicula, and on the back at the xiphoid level. Signal quality was checked by a calibration over 30 heartbeats. The calibration initially determines the SVi, computed with the subject during resting conditions. The measurement is based on the following formula for CO:

$$\mathrm{CO}\;(L/\min)=\mathrm{HR}\;(\mathrm{bpm})\times \mathrm{SVi}\;(\mathrm{mL}/m^{2})\times \mathrm{body}\;\mathrm{surface}\;\mathrm{area}\;(m^{2})$$

where body surface area is calculated according to the Haycock formula (32):

$$\mathrm{body}\;\mathrm{surface}\;\mathrm{area}\;(m^{2})=0.024265\times \mathrm{height}\;(\mathrm{cm}{)}^{0.3964}\times \mathrm{body}\;\mathrm{mass}\;(\mathrm{kg}{)}^{0.5378}$$

SVi is calculated as follows:

$$\mathrm{SVi}=k\times [dZ/dt_{\max}/(Z_{\max}-Z_{0})]\times W\;(\mathrm{TFIT})$$

The largest impedance variation reached during systole (*Z*_max_ − *Z*_0_) and the largest variation rate of the impedance signal (*dZ*/*dt*_max_) were used in the calculation [detailed information on the calculation can be found in Figure 1 of Charloux et al.'s publication (14)]. SVi also depends on the time of the ejection phase of the chamber described by the thoracic flow inversion time (TFIT). TFIT describes the time interval from the first zero value after the beginning of the cardiac cycle to the following minimum value after *dZ*/*dt*_max_, the maximal ejection velocity. *k* describes a constant value, and W(TFIT) is a weighted value of the TFIT using a special algorithm that includes two parameters additional to the electrical signal: HR and blood pressure difference (33).

In addition to CO, HR, and SVi, the PhysioFlow® provides several other hemodynamic parameters, including CI, VET, CTI, LCWi, SVRi, SVR, EDFR, EDV, and EF. The methods and calculations for deriving these additional parameters have been documented in detail by Gordon et al. (13).

Hemodynamic parameters were measured continuously from the start of the CPET until the cooldown phase. During the entire test procedure, the tester continuously monitored signal strength and quality. For hemodynamic values at P_VT1_, P_VT2_, and P_V̇O2peak_, the average over 30 s was calculated (15 s before and after threshold) and used for further analysis. Only signal qualities of the impedance cardiograph ≥95% were accepted.

### Statistical analysis

All data were analyzed using SPSS Statistics (Version 28, IBM, Armonk, NY, USA), and figures were created using GraphPad Prism (Version 9.3.1, GraphPad Software, San Diego, California, USA). No prior sample size calculation was performed for this secondary outcome, the analysis should be considered exploratory in nature, and results should be interpreted with caution. The differences in the variables between Test 1 and Test 2 were controlled for normal distribution using quartile–quartile plots (34). Values of *p* ≤ 0.05 were considered significant (two-sided *p*). Differences between Test 1 and Test 2 in hemodynamic parameters at P_VT1_, P_VT2_, and P_V̇O2peak_ were determined using paired *t*-tests. Test-retest reliability was assessed using the ICC. The lower limits of the CI_95%_ of the ICC were used to classify reproducibility: <0.50 was defined as poor reliability, 0.50–0.75 as moderate, 0.75–0.90 as good, and >0.90 as excellent reliability (35). Data are presented as mean and standard deviation (SD). Standard error of measurement (SEM) and minimal detectable change (MDC) were calculated for each parameter and threshold according to the following formulas: $\left(\mathrm{SEM}=\mathrm{SD}\sqrt{1-\mathrm{ICC}}\right)$ and $\left(\mathrm{MDC}=\mathrm{SEM}\times 1.96\times \sqrt{2}\right)$ (36). To evaluate the agreement or bias between Test 1 and Test 2, the Bland–Altman method (37) was used, where the difference between SV in Test 1 and Test 2 for three thresholds during incremental exercise testing (P_VT1_, P_VT2_, and P_V̇O2peak_) were plotted against the mean of the two tests. For the limit of agreement, the 95% tolerance interval with an 80% confidence level was chosen (38, 39).

Bland–Altman analysis can not only be used to compare a new measurement method with a gold standard but is also applicable for assessing test-retest reliability by analyzing the agreement of a single measurement method (37). It has also been utilized for this purpose in previous studies (22, 40).

The decision to focus on SV analysis was based on the CO formula: CO = HR × SV. Since HR can be reliably measured and remains relatively stable within a test-retest design (13), variations in CO are most likely attributable to differences in SV measurement. A Preiss–Fisher analysis was performed to validate the differences observed in the Bland–Altman analysis and to confirm that the measurement range was sufficiently wide (Supplementary Figure S1) (41, 42).

In addition, an *a priori* acceptable range for agreement was defined to account for possible variations in SV across different thresholds, while ensuring that V̇O_2_ variations remained within a tolerance of 4.0 mL/kg/min. This range was chosen based on the day-to-day variability of mean V̇O_2peak_ of 2.0 mL/kg/min with an SD of 1.0 mL/kg/min (25). Assuming a normal distribution, 68% of the population falls within ±1 SD, and 95% within ±2 SD. Therefore, the tolerance range was set at 4 mL/kg/min, derived as the mean (2.0 mL/kg/min) plus 2 times the SD (1.0 mL/kg/min). For this purpose, V̇O_2_, body weight, HR, and CO were analyzed separately for the women and men at each characteristic phase and threshold. According to the Fick principle [V̇O_2_ = HR × SV × arteriovenous oxygen difference (avDO_2_)] and assuming constancy in HR and avDO_2_, the potential variation in SV was calculated in the women and men separately. This separation was necessary because the V̇O_2peak_ values at each characteristic phase and threshold differ significantly between sexes, leading to different potential changes in SV. The acceptable range of agreement was incorporated into the Bland–Altman plots.

## Results

### Participants characteristics

Two participants had to be excluded due to the poor signal quality (<95%) resulting in insufficient data from the PhysioFlow® during either Test 1 or Test 2. This led to 29 participants (13 women, 16 men) with complete data for analysis (Figure 1). Participant characteristics are presented in Table 1. For assessment of reliability at different thresholds and characteristic phases, two measurements of hemodynamic parameters had to be excluded at P_V̇O2peak_ due to poor signal quality (<95%) from the PhysioFlow® for either Test 1 or Test 2. At P_VT1_ and P_VT2_, the PhysioFlow® provided sufficient signal quality in both tests for all participants.

**Table 1:** Characteristics of the study population (*n* = 29).

Variable	Total (*n* = 29)		Men (*n* = 16)		Women (*n* = 13)	
Age (years)	24	(3)	24	(2)	23	(3)
Body mass index (kg/m^2^)	22.1	(2.1)	22.7	(1.6)	21.4	(2.4)
V̇O_2peak_ (L/min)	3.74	(0.75)	4.31	(0.38)	3.03	(0.40)
V̇O_2peak_ (mL/kg/min)	57.7	(5.2)	61.1	(3.9)	53.6	(3.1)
P_VT1_ (Watt)	185	(45)	210	(39)	153	(30)
P_VT2_ (Watt)	301	(57)	341	(36)	251	(34)
P_V̇O2peak_ (Watt)	338	(62)	384	(35)	282	(35)

Data are mean (standard deviation).

P_VT1_, wattage at ventilatory threshold 1; P_VT2_, wattage at ventilatory threshold 2; P_V̇O_2_peak_, wattage at peak oxygen uptake; V̇O_2peak_, peak oxygen uptake.

### Reliability of hemodynamic parameters during incremental exercise testing

Statistical parameters (ICC, SEM, and MDC) for hemodynamic variables at P_VT1_, P_VT2_, and P_V̇O2peak,_ are presented in Table 2. HR showed good reliability as the lower limit of the CI_95%_ for the ICC was above 0.75 at all three exercise intensities investigated. In detail, the mean ICC at P_VT1_ was 0.91 (CI_95%_: 0.81; 0.96), at P_VT2_ 0.92 (0.84; 0.96), and at P_V̇O2peak_ 0.93 (0.85; 0.97). SV, CO, and EDV demonstrated good reliability for mean ICC, all of which were above 0.75 at all three time points. However, as the lower levels of the CI_95%_ for all three were <0.75 at all three time points, the reliability was classified as moderate. All other parameters showed poor reliability (<0.50) or inconsistent reliability across the different exercise intensities, with VET and EF even showing significant differences at P_V̇O2peak_ between Test 1 and Test 2.

**Table 2:** Test-retest reliability of hemodynamic parameters using the PhysioFlow® at P_VT1_, P_VT2_, and P_V̇O2peak_ during incremental exercise testing on a cycle ergometer.

Threshold	Parameter	Test 1		Test 2		Difference		ICC		SEM	MDC
		Mean	(SD)	Mean	(SD)	Mean	(SD)	Mean	(CI_95%_)		
P_VT1_ (*n* = 29)	HR (bpm)	146	(15)	144	(16)	−2	(7)	0.91	(0.81, 0.96)	2.01	5.56
	SV (mL)	108.3	(25.6)	107.0	(19.7)	−1.3	(14.5)	0.80	(0.61, 0.90)	6.53	18.09
	CO (L/min	15.8	(3.8)	15.4	(3.0)	−0.4	(2.3)	0.77	(0.57, 0.89)	1.11	3.07
	SVi (mL/m^2^)	62.0	(12.4)	61.4	(9.4)	−0.6	(8.2)	0.72	(0.49, 0.86)	4.30	11.92
	Ci (L/min/m^2^)	9.0	(1.8)	8.8	(1.5)	−0.2	(1.3)	0.69	(0.44, 0.84)	0.72	2.00
	VET (ms)	264.7	(41.1)	254.7	(42.4)	−10.0	(48.2)	0.33	(−0.03, 0.62)	39.31	108.97
	CTI	491.6	(107.3)	485.5	(129.0)	−6.1	(83.7)	0.75	(0.54, 0.88)	41.79	115.83
	LCWi (kg*m/m^2^)	11.5	(2.8)	11.1	(82.2)	−0.3	(2.0)	0.69	(0.44, 0.84)	1.10	3.04
	SVRi (dynes s*m^2^/cm^5^)	831.2	(163.1)	844.1	(175.1)	12.9	(135.5)	0.68	(0.42, 0.84)	76.77	212.81
	SVR (dynes s/cm^5^)	479.8	(93.9)	485.8	(93.9)	6.0	(76.8)	0.67	(0.40, 0.83)	44.46	123.24
	EDFR (%)	46.1	(9.3)	52.3	(24.6)	6.2	(23.7)	0.19	(−0.19, 0.51)	21.40	59.31
	EDV (mL)	134.3	(28.4)	136.2	(24.5)	1.9	(18.1)	0.77	(0.56, 0.88)	8.73	24.20
	EF (%)	80.6	(7.2)	79.0	(7.5)	−1.6	(5.0)	0.77	(0.56, 0.88)	2.43	6.74
P_VT2_ (*n* = 29)	HR (bpm)	179	(9)	178	(9)	−1	(3)	0.92	(0.84, 0.96)	0.92	2.56
	SV (mL)	108.6	(27.1)	108.2	(21.5)	−0.4	(15.5)	0.80	(0.62, 0.90)	6.95	19.28
	CO (L/min	19.5	(4.7)	19.2	(3.7)	−0.2	(2.9)	0.77	(0.57, 0.89)	1.38	3.82
	SVi (mL/m^2^)	62.2	(13.4)	62.1	(10.6)	−0.1	(8.7)	0.74	(0.51, 0.87)	4.47	12.40
	Ci (L/min/m^2^)	11.1	(2.3)	11.0	(1.8)	−0.1	(1.6)	0.69	(0.43, 0.84)	0.91	2.52
	VET (ms)	233.1	(25.3)	232.1	(29.9)	−1.0	(21.9)	0.69	(0.44, 0.84)	12.19	33.78
	CTI	470.9	(120.0)	487.2	(149.4)	16.2	(120.6)	0.60	(0.31, 0.79)	75.89	210.34
	LCWi (kg*m/m^2^)	14.2	(3.6)	13.9	(2.6)	−0.3	(2.3)	0.74	(0.51, 0.87)	1.17	3.23
	SVRi (dynes s*m^2^/cm^5^)	673.9	(119.3)	673.6	(113.1)	−0.4	(112.5)	0.53	(0.21, 0.75)	76.97	213.35
	SVR (dynes s/cm^5^)	389.8	(75.2)	388.9	(69.5)	−0.9	(63.8)	0.61	(0.32, 0.80)	39.73	110.13
	EDFR (%)	48.3	(10.6)	46.9	(10.9)	−1.4	(10.5)	0.53	(0.20, 0.75)	7.24	20.06
	EDV (mL)	136.0	(27.1)	137.5	(25.0)	1.5	(17.6)	0.77	(0.57, 0.89)	8.37	23.21
	EF (%)	79.6	(7.8)	78.9	(8.5)	−0.7	(7.3)	0.60	(0.30, 0.79)	4.64	12.87
P_V̇O2peak_ (*n* = 27)	HR (bpm)	187	(9)	186	(9)	−1	(3)	0.93	(0.85, 0.97)	0.89	2.47
	SV (mL)	109.6	(26.1)	108.6	(23.3)	−1.0	(14.9)	0.82	(0.64, 0.91)	6.31	17.50
	CO (L/min	20.5	(4.7)	20.2	(4.3)	−0.3	(2.8)	0.80	(0.61, 0.91)	1.26	3.51
	SVi (mL/m^2^)	62.9	(12.7)	62.2	(11.1)	−0.6	(8.6)	0.74	(0.50, 0.87)	4.43	12.28
	Ci (L/min/m^2^)	11.7	(2.2)	11.6	(2.0)	−0.2	(1.7)	0.69	(0.43, 0.85)	0.92	2.55
	VET (ms)	226.4	(27.2)	207.2	(39.4)	−19.2	(41.1)	0.27	(−0.12, 0.58)	35.22	97.63
	CTI	482.5	(119.1)	439.7	(132.0)	−42.8	(122.7)	0.52	(0.19, 0.75)	84.67	234.70
	LCWi (kg*m/m^2^)	14.8	(3.5)	14.6	(3.0)	−0.3	(2.4)	0.73	(0.50, 0.87)	1.23	3.41
	SVRi (dynes s*m^2^/cm^5^)	633.4	(96.5)	644.4	(105.6)	11.0	(99.1)	0.52	(0.18, 0.75)	68.66	190.32
	SVR (dynes s/cm^5^)	367.1	(62.9)	374.8	(80.5)	7.8	(61.4)	0.64	(0.35, 0.82)	36.89	102.26
	EDFR (%)	48.2	(12.2)	54.4	(19.2)	6.2	(17.8)	0.38	(0.01, 0.66)	14.01	38.83
	EDV (mL)	136.9	(28.1)	141.6	(29.1)	4.7	(19.9)	0.76	(0.54, 0.88)	9.82	27.23
	EF (%)	80.0	(6.8)	76.8	(7.5)	−3.1	(5.9)	0.66	(0.38, 0.83)	3.45	9.58

Data are mean (SD).

HR, heart rate; SV, stroke volume; CO, cardiac output; SVi, stroke volume index; Ci, cardiac index; VET, ventricular ejection time; CTI, contractility index; LCWi, left cardiac work index; SVRi, systemic vascular resistance index; SVR, systemic vascular resistance; EDFR, early diastolic filling ratio; EDV, end diastolic volume; EF, ejection fraction; P_VT1_, wattage at ventilatory threshold 1; P_VT2_, wattage at ventilatory threshold 2; P_V̇O2peak_, wattage at peak oxygen uptake; SD, standard deviation; CI_95%_, 95% confidence interval; ICC, intraclass correlation coefficient; SEM, standard error of measurement; MDC, minimal detectable change.

**Figure 2:**
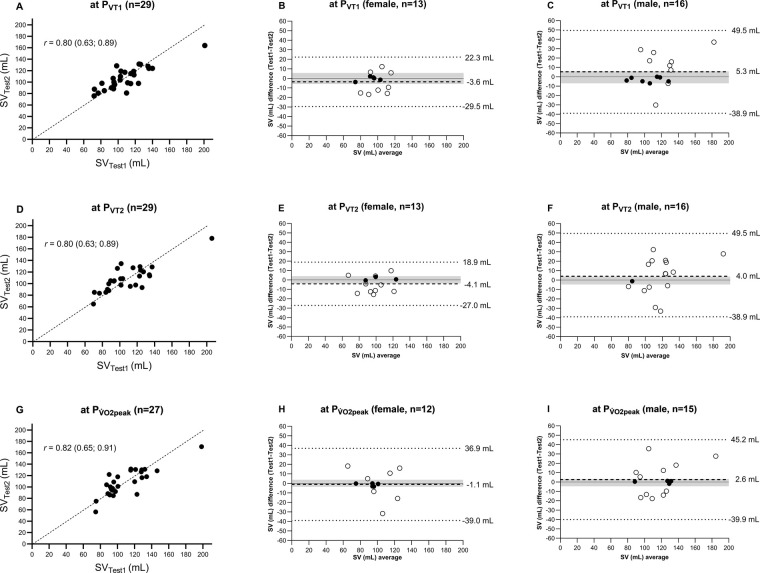
Comparison of SV measured at P_VT1_, P_VT2_, and P_V̇O2peak_ during incremental exercise testing between Test 1 and Test 2. **(A,D,G)** SV_Test1_ vs. SV_Test2_ with the line of identity and Lin's concordance correlation coefficient. **(B,E,H,C,F,I)** Mean values and differences in mean (dashed line), 95% limits of agreement with 80% confidence interval (dotted line), and *a priori*-defined acceptable range of data (grey area) for female and male participants, respectively. SV*,* stroke volume; *P*_VT1_, wattage at ventilatory threshold 1; P_VT2_, wattage at ventilatory threshold 2; P_V̇O2peak_, wattage at peak oxygen uptake.

The SV measurement values at P_VT1_, P_VT2_, and P_V̇O2peak_ in Test 1 and Test 2 are plotted in Figures 2(A,D,G). Lin's concordance correlation coefficient showed moderate correlation at each of the three exercise intensities investigated [P_VT1_: 0.80 (0.63; 0.89), P_VT2_: 0.80 (0.63; 0.89), and P_V̇O2peak_: 0.82 (0.65; 0.91)].

Figures 2(B,C,E,F,H,I) provide a Bland–Altman comparison of Test 1 and Test 2. For the female participants, the limits of agreement (95% tolerance interval with 80% confidence level) for SV at P_VT1_, P_VT2_, and P_V̇O2peak_ were −29.5 to 22.3, −27.0 to 18.9, and −39.0 to 36.9 mL, respectively. For the male participants, the limits of agreement were wider for each threshold with −38.9 to 49.5 mL at P_VT1_, −38.9 to 49.5 mL at P_VT2_, and −39.9 to 45.2 mL at P_V̇O2peak_. All measurements were within the limits of agreements for all thresholds for both the women and men.

*A priori* defined acceptable range of agreement for SV were −5.9 to 5.6 mL at P_VT1_, −3.7 to 4.0 mL at P_VT2_, and −3.6 to 3.8 mL at P_V̇O2peak_ for the women and −7 to 5.8 mL at P_VT1_, −4.7 to 3.6 mL at P_VT2_, and −4.5 to 3.3 mL at P_V̇O2peak_ for the men.

For the women, 38% (*n* = 5 out of 13 measurements) were within the range at P_VT1_, 23% (*n* = 3) at P_VT2_, and 42% (*n* = 5) at P_V̇O2peak_. For the men, 44% (*n* = 7) at P_VT1_, 6% (*n* = 1) at P_VT2_, and 25% (*n* = 4) at P_V̇O2peak_.

## Discussion

The major novel finding is that during an incremental ramp test, the reliability was good for HR, SV, CO, and EDV at the group level (mean ICC) at VT1, VT2, and V̇O_2peak_. This is in line with Gordon et al. (13), where reliability was assessed at various exercise intensities under steady-state conditions for 5 min at 50% and 70% of peak power output and 30-s intervals at 90% of peak power output. The ICC (CI_95%_) for SV for the 50% steady-state, 70% steady-state, and intervals were 0.85 (0.60; 0.94), 0.91 (0.78; 0.97), and 0.87 (0.83; 0.90). Similar results were obtained by Schultz et al. (22) in older participants (57 (9) years), showing an ICC for SV of 0.69 during steady-state cycling at 60% of HR_max_ and an ICC of 0.90 at 70% of HR_max_. Legendre et al. (17) investigated the reliability of the SVI in patients with residual right outflow tract lesions after congenital heart disease repair, reporting an ICC of 0.80, also in a comparable range. In addition, a study in children reported an ICC of SV at V̇O_2peak_ of 0.88 (CI_95%_: 0.76; 0.94) (18).

However, the classification of reliability based on mean ICC alone is insufficient, as illustrated in Figure 2. The analyses showed that a change in SV, for instance, at P_V̇O2peak_, from −3.6 to 3.8 mL in the women and −4.5 to 3.3 mL in the men, led to an alteration in V̇O_2peak_ of approximately ±2.0 mL/kg/min. Knaier et al. (25) demonstrated that a change of 2.0 ± 1 mL/kg/min could occur due to day-to-day variations. This implies that changes exceeding this range likely indicate a “real” physiological change. The MDC of 17.5 mL (Table 2) confirms that this variation (−3.6 to 3.8 mL in the women, −4.5 to 3.3 mL in the men) lies beyond the detection capabilities of the PhysioFlow®. Therefore, despite similar findings in previous studies, the device's reliability is classified as moderately reliable.

This issue is also reflected in the limits of agreement in the Bland–Altman plots. For example, differences in measured SV at P_V̇O2peak_ are contained within a 95% tolerance interval at an 80% confidence level, ranging from −39.0 to 36.9 mL for the women and from −39.9 to 45.2 mL for the men. This indicates that individual measurements can vary significantly upon repeated measurements. Thus, while the SV measurements are reliable at the group level (the mean difference between Test 2 and Test 1 at P_V̇O2peak_ was −1.0 (14.9) mL), they fail to provide good reliability for individual assessments. The same conclusion also applies to the measurement of SV at P_VT1_ and P_VT2_.

All other hemodynamic parameters (SVi, Ci, VET, CTI, LCWi, SVRi, SVR, EDFR, and EF) provided by the PhysioFlow® demonstrated poor or inconsistent reliability across the thresholds and cannot be used either at the group or individual level. This result differs from that of Gordon et al. (13), as they reported a mean ICC greater than 0.75 for LCWi under steady-state exercise at various intensities.

Compared to the study of Gordon et al. (13), a similar percentage of participants had to be excluded due to insufficient signal quality during maximal exercise [7% (*n* = 2) vs. 5% (*n* = 1) at 90% peak power output during 30-s intervals], but a lower percentage than reported by Kemps et al. (43) [24% (*n* = 24) of all measurements during exercise]. Schultz et al. (22) also examined the reproducibility of SV during steady-state exercise using impedance cardiography. They excluded 14% (*n* = 7) at 40 W, 61% (*n* = 31) at 60% HR_max_, and 47% (*n* = 24) at 90% HR_max_ due to “unavailability of data at one of the visits.” The present data demonstrated that sufficient signal quality was achieved with increasing workload up to P_VT2_; thus, in contrast to Schultz et al. (22) no data were lost, and at P_V̇O2peak_, only 7% (*n* = 2) had to be excluded due to insufficient signal quality. The exact reasons for the differences could not be identified. Kemps et al. (43) suspected that qualitatively insufficient measurements were caused by an irregular or oscillating breathing pattern in their study population of patients with chronic heart failure. Gordon et al. (13) provided no reasons for insufficient signal quality. In this study, it is suspected that movement and respiratory artifacts could be the reason for insufficient signal quality. Notably, Warburton et al. (9) have previously identified these factors as potential limitations in using impedance cardiography during maximal exercise. Another explanation could be increased sweat production, which can influence impedance signals (44), as they only occurred during maximum load and not before.

### Limitation

Based on current knowledge, this is the first study to investigate the reliability of the PhysioFlow® at the VTs during CPET in well-trained adults. Another innovation was the *a priori-*defined acceptable range of agreement of SV depending on the resulting clinically relevant difference in V̇O_2_.

Since this study exclusively examined the test-retest reliability of a single measurement method for assessing hemodynamic parameters and did not include comparisons with the direct Fick method, any assertions concerning validity are excluded. It should be noted that participants in the current study were young, healthy, and showed high endurance performance levels. Therefore, the results of this study are not representative of other population groups.

The statistical power of this study was primarily calculated for a different primary outcome, which may limit the ability to detect clinically relevant differences in this secondary outcome. As no specific power analysis was conducted for this research question, the findings should be considered exploratory in nature and interpreted with caution.

Signal quality issues occurred in 7% of measurements, but the underlying causes could not be determined.

## Conclusion

This study was the first to assess the test-retest reliability of the PhysioFlow® at characteristic phases and thresholds in healthy, well-trained adults during CPET. These findings demonstrate that the PhysioFlow® can reliably measure HR, SV, CO, and EDV at the group level during incremental exercise testing and has limited ability to capture individual differences accurately. This finding is crucial for clinical applications, highlighting that while the device effectively measures SV for broader, group-based assessments, it may not be suitable for precise individual diagnostics without considering potential variability.

## Data Availability

The raw data supporting the conclusions of this article will be made available by the authors, without undue reservation.

## Supplementary material

Supplementary Figure S1 Preiss-Fisher analysis for validation of measurement differences: confirming normal distribution in Bland–Altman method results. VT1, ventilatory threshold 1; VT2, ventilatory threshold 2; V̇O_2peak_, peak oxygen consumption.
