# Supplementary figures and images for: Reliability of hemodynamic parameters measured by bioimpedance cardiography at different intensities during incremental exercise testing
Source: Front Cardiovasc Med. 2025 Apr 10;12:1531027. doi: 10.3389/fcvm.2025.1531027 (PMC12018426; doi:10.3389/fcvm.2025.1531027)

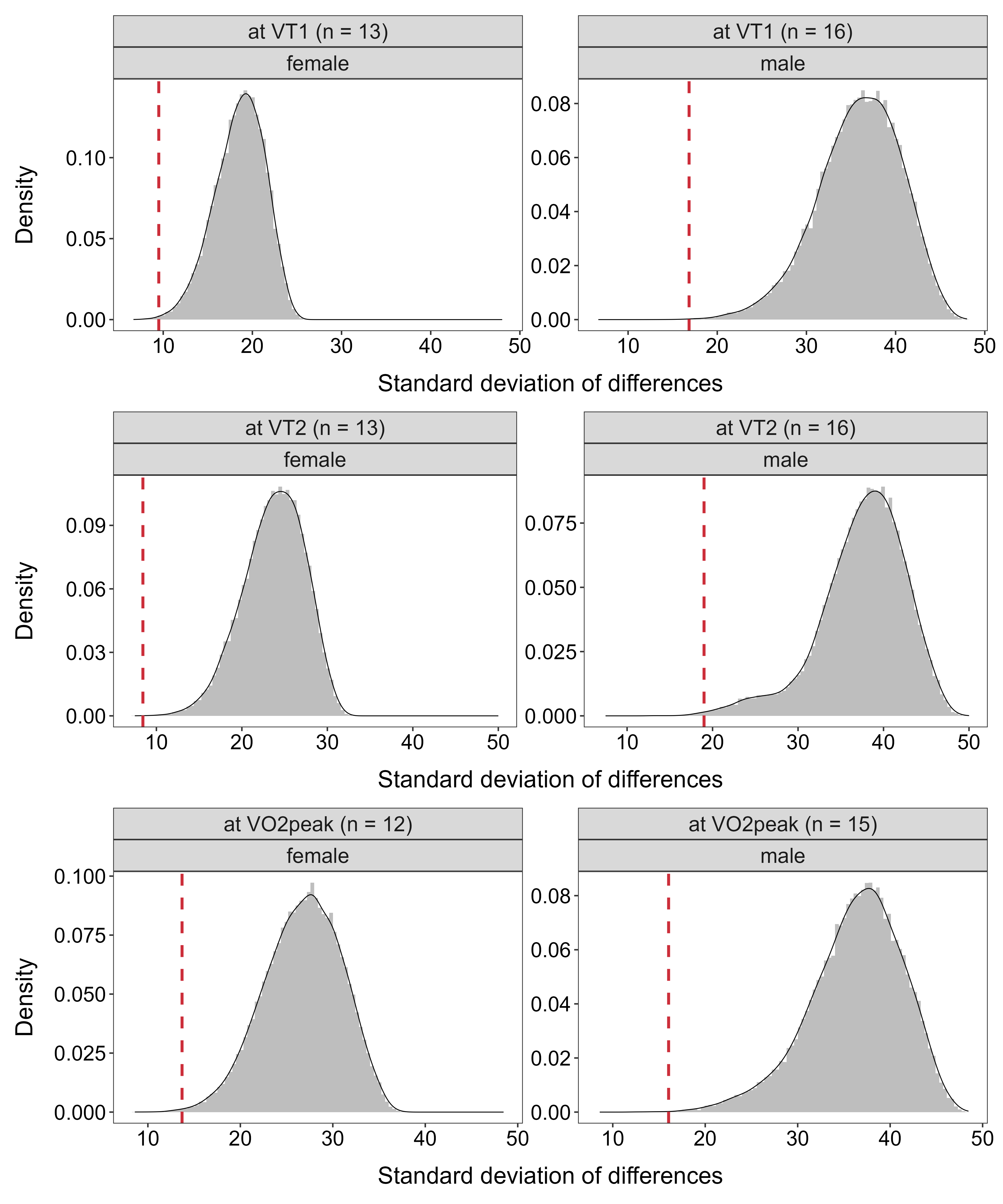

Supplement: Supplementary Figure S1 — Preiss-Fisher analysis for validation of measurement differences: confirming normal distribution in Bland–Altman method results. VT1, ventilatory threshold 1; VT2, ventilatory threshold 2; V̇O2peak, peak oxygen consumption. [file Image1.png]
